# Single-Chain Fragment Variable: Recent Progress in Cancer Diagnosis and Therapy

**DOI:** 10.3390/cancers14174206

**Published:** 2022-08-30

**Authors:** Paola Muñoz-López, Rosa María Ribas-Aparicio, Elayne Irene Becerra-Báez, Karla Fraga-Pérez, Luis Fernando Flores-Martínez, Armando Alfredo Mateos-Chávez, Rosendo Luria-Pérez

**Affiliations:** 1 Unit of Investigative Research on Hemato-Oncological Diseases, Hospital Infantil de México Federico Gómez, Doctor Márquez 162, Mexico City 06720, Mexico; 2 Departamento de Microbiología, Escuela Nacional de Ciencias Biológicas, Instituto Politécnico Nacional (IPN), Prolongación de Carpio y Plan de Ayala S/N, Mexico City 11340, Mexico; 3 Departamento de Bioquímica, Escuela Nacional de Ciencias Biológicas, Instituto Politécnico Nacional (IPN), Prolongación de Carpio y Plan de Ayala S/N, Mexico City 11340, Mexico

**Keywords:** single-chain fragment variable, cancer therapy, immunotherapy

## Abstract

**Simple Summary:**

Recombinant antibody fragments have shown remarkable potential as diagnostic and therapeutic tools in the fight against cancer. The single-chain fragment variable (scFv) that contains the complete antigen-binding domains of a whole antibody, has several advantages such as a high specificity and affinity for antigens, a low immunogenicity, and the proven ability to penetrate tumor tissues and diffuse. This review provides an overview of the current studies on the principle, generation, and applications of scFvs, particularly in the diagnosis and therapy of cancer, and underscores their potential use in clinical trials.

**Abstract:**

Cancer remains a public health problem worldwide. Although conventional therapies have led to some excellent outcomes, some patients fail to respond to treatment, they have few therapeutic alternatives and a poor survival prognosis. Several strategies have been proposed to overcome this issue. The most recent approach is immunotherapy, particularly the use of recombinant antibodies and their derivatives, such as the single-chain fragment variable (scFv) containing the complete antigen-binding domains of a whole antibody that successfully targets tumor cells. This review describes the recent progress made with scFvs as a cancer diagnostic and therapeutic tool, with an emphasis on preclinical approaches and their potential use in clinical trials.

## 1. Introduction

**Figure 1:**
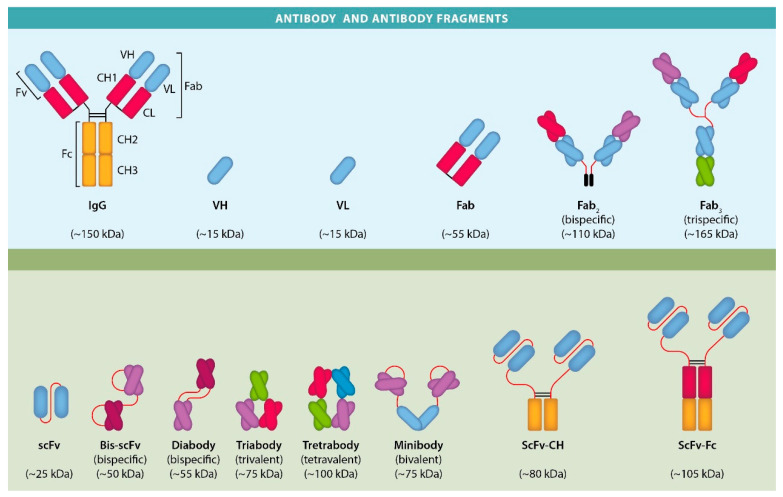
The schematic representation of an antibody and its derivatives. A classic molecule of an IgG antibody is shown with the different fragments obtained from the antibody through genetic engineering.

As stated by the World Health Organization (WHO), cancer is one of the major causes of death worldwide. In 2020, approximately 10 million deaths were estimated, and global calculations have shown that 1 in 6 deaths are the result of cancer [1]. Although conventional therapies have led to excellent outcomes, some patients fail to respond to treatment, they have few alternative therapies and a poor survival prognosis. Given this scenario, the development of modern methods for the early detection and adequate treatment of cancer is essential. Over a century after Paul Ehrlich hypothesized the use of “magic bullets” against different pathologies, antibodies have transformed this hypothesis into a real treatment against cancer [2]. Progress in immunotherapy as a therapeutic alternative against cancer has allowed the use of antibodies and their derivatives as diagnostic and therapeutic tools [3]. Much effort has been devoted to the improvements in the design and production of these antibodies and their derivatives, using more efficient and affordable platforms. Investigators have undertaken the task of searching for alternatives to quandaries that have developed, including the construction of Fab fragments [4], fragment variable (Fv) in which variable domains (V) are linked by non-covalent forces [5], and the so-called single-chain fragment variable (scFv) [6]. (Figure 1). 

In 1988, Houston et al., designed a single-chain antibody with the variable domain of the 26-19 anti-digoxin monoclonal antibody; this scFv consisted of a single polypeptide connected with a 15 amino acid linker. The final protein was expressed in *Escherichia coli (E. coli)* and the peptide linker, Asp-Pro, was obtained by acid excision. The linker joined the signaling sequence and the scFv ((signaling sequence)-Asp-Pro-VH-(linker)-VL). After its isolation and renaturing, the scFv showed clear specificity against digoxin and related cardiac glycosides that were similar to the native Fab 26-10 fragments [7]. 

Later, Cheadle et al., cloned and expressed the Fv portion of MOPC315, a mouse myeloma protein; the recombinant chains initially recovered as inclusion bodies, they then dissolved and combined with each other. The Fv fragment was biologically active and had an identical affinity as that of the native Fv. The activity of the recombinant MOPC315 Fv was also obtained with an scFv in which the recombinant VH and VL were connected with a (Gly4Ser)3 linker, proving that the binding activity of scFvs was the same as that of native and recombinant Fvs [8]. Based on those studies, scFvs have become very relevant and have preclinical, clinical, and research applications. Advances in antibody engineering have made the generation of highly personalized scFvs possible, with improved pharmacokinetic properties and hence, with much greater clinical relevance.

The scFvs are molecules with a rational polypeptide design that only consists of the variable regions of heavy (VH) and light (VL) chains connected by a linker. They maintain the binding specificity of the original antibody and possess various advantages over full-length monoclonal antibodies, including: the abrogation of undesirable side effects conditioned by the Fc portion of the complete antibody, they are easily constructed and expressed, and they can be produced on a large scale. scFvs also have improved pharmacokinetic features, such as a greater rate of penetration in blood vessel walls and solid tumors, so they are the preferred vehicle for directed drug delivery and may also be beneficial when applied in radiotherapeutic and diagnostic methods [9]. In addition, scFvs cause few or no hypersensitivity or rejection reactions and may act as carriers of encapsulated drugs or treatment proteins. Finally, the scFv is rapidly cleared in vivo, thus decreasing possible injury to adjacent tissues since their average life span is 0.5–2 h. These characteristics prove that scFvs play an important role in cancer diagnostics and therapeutic [10,11], and they currently account for close to 35% of all antibody fragments under scrutiny in clinical trials [12,13].

We herein describe recent progress in harnessing scFvs as diagnostic tools and cancer therapy, emphasizing preclinical approaches and their potential use in clinical studies.

## 2. Single-Chain Fragment Variable (scFv) Engineering

### 2.1. General Properties of scFv

Functionally, an antibody is comprised of three components: a crystallizable fragment (Fc) and two antigen-binding fragments (Fab). The first binds to a number of cells and this determines its interactions with other components of the adaptive immune system. The second and third include the coupling of the variable domains of the heavy and light chains (VH and VL); each pair of variable domains creates a so-called variable fragment (Fv) (Figure 1) [14,15]. The Fvs are small in size, thus conferring certain advantages over a complete antibody, such as a better tissue and tumor penetrability and faster elimination from the body. However, these fragments show low stability since their two units, VH and VL, are easily dissociated and their antigen affinity is often lower in comparison with the parental antibody. Several strategies have been developed to overcome these difficulties and among them is the purification of the scFvs [16,17]. It is constituted by the VH and VL chains connected by a flexible polypeptide linker weighing 25–30 kDa and it maintains the same specificity and affinity for antigens without a need for the constant region [18]. The simple structure of scFvs eliminates the complications resulting from the use of complete antibodies and they have shown a superior capacity to penetrate tissues and diffuse within them (six times faster than immunoglobulin G) [19,20,21]. Both VH-linker-VL and VL-linker-VH have been successful as scFvs [22], and the VH and VL domains are linked together with a peptide sequence and can be expressed as a single protein.

Fragment stability is essential to guarantee all scFvs applications in vitro and in vivo [23]. Unfortunately, many scFvs show little stability and only for a few hours or days after their experimental incorporation into human serum. The lack of stability of the VH–VL domains of scFvs is often the main reason underlying their lack of functionality and their tendency to aggregate [24]. Several studies have examined the effects of different linker designs on scFv properties.

The length and content of the linker sequence are two features that may affect the degree of expression, folding, the oligomeric state, affinity and specificity, and the in vivo stability and activity of the scFvs [25,26,27]. If the scFv linker length is greater than 12 residues, the covalently linked VH and VL create a highly monomeric and very stable functional scFv [28]; scFvs with shorter linkers (<12 aa) tend to form multimers when combined with other scFv molecules, and scFvs with 15–20 amino acid linkers have shown the best binding properties. A ~15-amino acid sequence [29] has been described as ideal for correct folding, and it should encompass 3.5 nm (35 Å) between the C-terminus of one of the variable domains and the N-terminus of the other domain. This design is more thermodynamically stable and the capacity of the domains to fold and maintain the antigen-binding site remains unchanged [7]. The linker´s composition should be thoroughly considered since a hydrophilic sequence appears to be ideal. The most popular designs include sequences with glycine and serine residues (Gly4Ser3), since these confer the necessary flexibility to correctly promote the orientation of both domains. Charged amino acids, such as lysine and glutamic acid, can also be added to increase solubility [30,31].

Within each of the two variable domains of scFv, there are three hypervariable or complementary determining regions (CDR) interlinked by framework regions (FR). Whereas the CDR is responsible for antigen-binding and its structure is complementary to the epitope, the remaining variable domains (FR) act as a scaffold with minimal variability when compared with CDR. Surprisingly, the contribution of every CDR to antigen binding varies. For instance, the CDR3 of the heavy chain plays a pivotal role by contributing 29% to linkage specificity, while CDR2L only contributes 4% of the specificity [32].

In general, monoclonal antibodies must have affinities above 1 nM to be considered for clinical development [33,34]. In the case of scFvs, several reports have documented improved affinity; for example, Boder et al., developed a mutant scFv with an affinity close to Kd = 48 fM and slower dissociation kinetics (median >5 days) than those of the streptavidin–biotin complex [35]. Current strategies used to optimize the affinity of scFvs have improved them 13.58-fold [36,37].

### 2.2. Generation and Expression of scFvs

The scFvs are mostly constructed in hybridomas, but the generation of a recombinant fragment from an existing monoclonal antibody requires additional genetic engineering technical modifications: cloning of the variable regions of an antibody, assembling the VH and VL regions to form the scFv, their cloning in expression vectors and the evaluation of the expression, purification, and functional study of the soluble recombinant fragment. Isolation of the VH and VL genes of the mAb is essential to the generation of recombinant fragments. In addition, V genes are amplified by PCR from complementary DNA (cDNA) obtained from its hybridoma. To do so, oligonucleotides that hybridize with the conserved ends are used to amplify most of the V genes and they contain restriction sites that facilitate manipulation. The cloning process and its evaluation must be rapidly performed due to the potential heterogeneity of the V sequences in the producing hybridoma [16,38].

The applications of the scFvs obtained with hybridoma techniques carry the same limitations than those observed when using complete antibodies of murine origin, whereby their repeated administration may lead to a human anti-mouse antibody (HAMA) reaction that in turn, can trigger severe secondary effects such as anaphylactic shock [39]. The expression of scFv in phages (phage display) is a technique that allows the generation of completely human antibody fragments, thus precluding HAMA-mediated side effects. Since this approach is based on an in vitro selection process, it does not depend on animal immunization and its associated limitations, and it can use completely human gene reservoirs. scFv expression in phages is based on the studies by Georg P. Smith on the filamentous phages that infect *E. coli*, the most widely characterized organism in terms of genetics and physiology [40]. He described which exogenous DNA fragments can fuse with the gene encoding protein pIII in a non-lytic filamentous phage and be expressed as a fusion protein on the virion´s surface [41]; this finding was further delved into by McCaffrey et al., who reported that a scFv can be successfully expressed on the phage surface as a functional protein and still maintain its antigen-binding capacity [42]. 

Cloning the genes encoding VH and VL in phagemids designed to express VH and VL as a scFv fused with protein pIII of the capsid of an *E. coli* filamentous bacteriophage, leads to the subsequent transformation of *E. coli* with this construct and the addition of an auxiliary phage, permits the creation of a phage scFv antibody library [43]. Depending on the origin of the V gene repertoire, there are three kinds of libraries: immune, naïve, and synthetic libraries. The immune libraries are constructed with cells obtained from convalescent patients or immunized donors (animal or human). This type of library is mainly used to obtain an antibody against a particular target. Its advantage lies in the fact that the V genes harbor hypermutations and already possess mature affinity for the target antigen [44]. Naïve libraries are cloned from reordered V genes obtained from non-immunized donor B cells (IgM), so as to isolate antibody fragments that bind to practically all antigens without the need for immunization or patient blood [45]. They are therefore very useful in the production of antibody fragments that are difficult to generate with hybridoma techniques, particularly against non-immunogenic or toxic antigens. In general, the affinities of scFvs isolated from small naïve libraries are lower than those isolated from larger libraries. This is why the size of the library is important when selecting high-affinity fragments [46]. Finally, synthetic libraries are created by combining gene sequences from the germinal line in conjunction with random complementary determinant regions (CDR) that are responsible for antigen binding. Most synthetic human antibody libraries are centered on the randomization of the CDR3 regions that tend to be more diverse and essentially responsible for antigen binding. Thus, libraries should be broadly selected in order to create high-affinity monoclonal antibodies [22].

Once the phage library is generated, the process of scFv selection (panning) relies on the immobilization of antigenic targets on a solid surface such as plastic surfaces, polystyrene tubing, microtiter plate wells [47,48], affinity chromatography column matrices [49], or magnetic beads. After incubating the phage library with the immobilized antigens, phages that do not bind or bind weakly are eliminated with a rigorous wash. Then, the bound phage antibody is eluted by trypsinization or by changing the pH and is used to infect *E. coli* [50]. The *E. coli* carrying the phagemid is then used to produce new phage particles that contain the antibody fragments. Usually, 3 to 5 panning rounds are necessary before the final selection. The antigen-specific scFvs are identified with a selection process using soluble monoclonal antibodies or monoclonal antibody phages and finally, detection is performed by ELISA [51] or flow cytometry [52]. 

Some advantages of the phage display technique are the generation of specific antibody fragments and the personalization of their functions [53,54]. In addition, during the selection procedures, phage display can also be applied to isolate proteins, catalyzers, or well-folded stable peptide substrates [55]. Importantly, phages are more stable and can be stored for several years at 4 °C [56]. Mutants with a higher affinity can also be generated through targeted mutagenesis [57,58].

Aside from the phage display technology, the ribosome display methodology also provides an advantage since the antibody production system does not depend on the use of cells or phages, that sometimes hamper the process. It has greater potential than the phage display when producing antibody libraries. This technology is based on the in vitro synthesis of monoclonal antibody fragments through ribosomes. The first step in this technology is the isolation of antibody genes from human cells. These genes are replicated through a standardized PCR procedure to obtain a large amount of antibody genetic material. The transcription of the isolated DNA is the following step, and messenger RNA (mRNA) molecules are incubated with isolated ribosomes. mRNA reading by the ribosomes leads to the synthesis of antibody fragments and the creation of a complex that includes the ribosome, the mRNA, and the antibody. This complex is stabilized in vitro, and when selecting the desired antibody, the complex comes into contact with the corresponding target so that the specifically recognized antibody is isolated. The isolated antibody not only carries the ribosome but also the mRNA encoding it, which is used in RT-PCR to obtain the gene sequence for successive cloning. This procedure allows the generation of extensive amounts of ribosome–mRNA–antibody complexes, creating large collections of different antibodies. Both procedures have created antibody libraries encompassing over one hundred trillion different human antibodies. This ensures the isolation and production of antibodies for libraries and against almost any antigen [59].

Alternatively, scFvs can also be created by sequential cloning [60,61] or combinatorial infections [62]. Several scFvs have been developed against haptens [63], proteins [60], carbohydrates [64,65], tumor antigens [66,67], and viruses [68], among others. To date, scFvs have been successfully isolated and have displayed well in a number of systems, including mammal and yeast cells [69], plant [70], and insect cells [71]; however, their ability to fold and release scFvs depends on the display system.

As previously described, the bacterial expression system most frequently used in scFv production is *E. coli.* The fermentation and genetic modification processed with recombinant DNA technology have been extensively developed. They yield good folding properties and foster fast-growing cultures [72,73,74] with a high yield rate (10–30% of the total cell protein) [75]. This is how multiple formats of antibody fragments have been successfully produced in such microorganisms: as Fab fragments [76], individual VH fragments [77], Fv fragments [5], and scFvs [78].

The antibody fragments can be expressed in various *E. coli* compartments, mainly as cytoplasmic inclusion bodies that subsequently must undergo in vitro renaturation; the correct folding of the functional protein is improved through the adequate rearrangement of the disulfide bond [79]. The periplasmic space is another expression compartment. According to F. Baneyx [72], the periplasmic space is characterized by containing proteins as chaperones and disulfide isomerases that promote the correct folding of recombinant proteins [76,80]. Finally, the antibody fragments can also be expressed on the outer membrane and/or outside the bacterium, if one or more signal sequences are added [79], such as outer membrane protein A (OmpA), pectate lyase B (PelB), or the new lipoprotein A (NlpA) [81].

To improve the affinity of the scFv against the antigen of interest, researchers have suggested several possibilities such as the generation of fragments that are more resistant to heat denaturation [82], the creation of rational designs of the fragments to prevent aggregation [27,83], the inclusion of some fusion proteins to improve solubility [84], and the coexpression of some chaperone molecules to ameliorate stability and binding activity [85,86]. Finally, fragment multimerization can be considered; diabodies or tribodies not only generate bivalence or trivalence, but they also improve Fv stability via the linkage design [87,88]. 

As a result of the single-chain configuration, bi-specific antibody fragments may be generated by connecting two scFvs with a linker; these molecules are bivalent with a valence for each antigen and with a size ranging between 50 and 60 kDa. The bispecific tandem scFv format has been widely used in cancer immunotherapy to redirect T lymphocytes toward tumor cells or cells associated with the tumor microenvironment. This format is the basis of the bispecific T-cell engagers (BiTE) [89,90,91].

Diabodies (Db) are bivalent molecules with two chains, each including a VH and a VL domain, either from the same or from a different antibody, and the two variable domains are connected by a short linker, usually composed of five residues, such as GGGGS. Since the linker´s length is shorter than required to permit intrachain assembly of an antigen-binding site, the variable regions of the different chains associate with each other to form a dimeric complex with two antigen-binding sites, resulting in a compact molecule with a molecular mass similar to that of a tandem scFv (∼50 kDa) (Figure 1) [92].

## 3. Applications of scFvs

**Figure 2:**
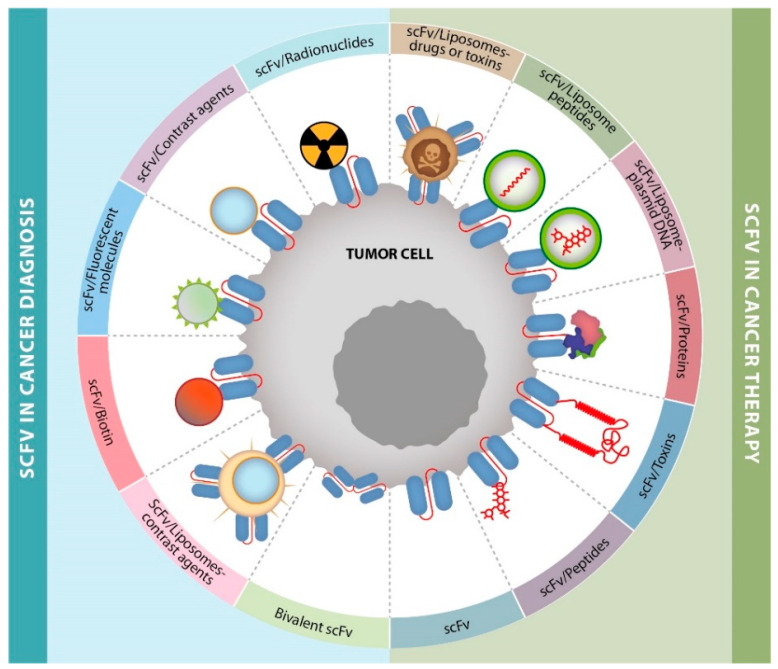
ScFv applications in cancer diagnosis or therapy. Specific scFvs have been used for diagnosis when coupled with: (A) radioisotopes, (B) contrast agents, (C) fluorescent molecules, (D) biotin, and (E) scFvs conjugated to liposomes loaded with contrast agents. Specific scFvs have also been used with therapeutic purposes for the delivery and/or release of several molecules, such as: (H) peptides, (I) toxins, (J) proteins, (K) plasmid-loaded liposomes, (L) peptide-loaded liposomes, and (M) scFvs conjugated to liposomes loaded with chemotherapy agents.

According to the WHO, cancer remains a very serious public health challenge [1], and the development of modern techniques for its early detection and adequate treatment are essential. Among these techniques, the use of scFvs has garnered great interest as a key tool in cancer therapies, diagnosis, and research [10,11,93], Figure 2. 

### 3.1. Cancer Diagnostics

Since antibodies can bind to a target molecule with high specificity and affinity, they can be used as immunological reagents in biomedical and clinical research. The detection of tumor cells has greatly improved in cancer, so adequate treatment and patient follow-up have also benefited.

Creating reagents for non-invasive diagnosis/prognosis with molecular imaging technologies such as positron emission tomography (PET) and magnetic resonance imaging (MRI), can improve the strategies for more effective treatments and improve cancer patient survival. Additionally, molecular imaging can also be used to monitor changes in tumor biology at different times throughout its course, and to analyze the pre- and post-treatment patient and tumor status. In this context, scFvs have a great ability to efficiently penetrate solid tumors due to their size and rapid clearance from the blood [21]. They are combined with fluorescent molecules or radioisotopes, thus becoming highly useful reagents.

The purification and characterization of scFvs against various tumor markers have been investigated for potential diagnostic use. Antibodies against classic biomarkers have been studied, and are still being analyzed in terms of biodistribution, their ability to penetrate tissue, toxicity, and efficient clearance. Some examples include scFvs directed against chondroitin sulfate (CS, abundant in the extracellular matrix in ovarian cancer) [94], CD24 (overexpressed in several tumors and in cancerous stem cells) [95], oncoprotein E6, expressed continuously in carcinomas associated with human papilloma virus type 16 (HPV 16) [96], gastrin receptor (CCK2R, playing a key role in the trigger and development of cancer) [97], dehydroepiandrosterone (DHEA, adrenocortical carcinoma marker) [98], against cancer cells overexpressing EGFR (epidermal growth factor receptor) [99], MCF-7 breast cancer cells [100,101], and SUM159 (breast cancer stem cells) [102]. They have all shown promising results.

The specificity of several scFvs has already been proven and they have been marked for visualization using molecular imaging techniques. Some have only been analyzed in vitro by ELISA, immunohistochemical staining, cell viability counts, confocal microscopy, flow cytometry, and immunofluorescence, among other assays. Examples of these include those that are specific against prostate stem cell antigen (PSCA) labeled with Alexa Fluor 647 [103], and against carcinoembryonic antigen (CEA), a blood and tumor tissue marker bound to enhanced green fluorescent protein (EGFP) [104]. Researchers have also created a scFv with affinity to the epithelial cell adhesion molecule EpCAM, that is overexpressed in most adenocarcinomas and squamous cell carcinomas [105]. It was bound to maleimide-DTPA (as a chelating agent) and gadolinium (Gd) to create the anti-EpCAM-Gd-DTPA and proved to be an effective in vitro MRI contrast agent, specifically in the diagnosis of colorectal cancer and other EpCAM-positive tumors.

Recently, a scFv against EGFR was developed and conjugated with Fe_3_O_4_/Au nanoparticles to create a bioprobe of a specific molecular magnetic resonance (scFv-Fe_3_O_4_/Au) to better identify EGFR-positive non-small cell lung cancer (NSCLC). The accumulation of this bioprobe in the tumor tissue was detected with MRI at certain time points after its systemic injection. Additionally, analysis with transmission electron microscopy (TEM) showed the specific location of scFv-Fe_3_O_4_/Au in the tumor cell cytoplasm. This proves that scFv-Fe_3_O_4_/Au could be a useful probe in the non-invasive diagnosis of EGFP-positive NSCLC [106].

Some scFvs have already been explored in vivo, such as those against tissue factor (TF), a trigger of the extrinsic coagulation cascade and that is overexpressed in several solid tumors (gastric, pancreatic, and brain) [107,108]. Accordingly, a specific scFv against TF was created and labeled with Alexa fluor 647 to test its usefulness as a probe in mice with cutaneous, chemically induced tumors [109]. The anti-TF scFv accumulated rapidly and specifically after 1–3 h. and was eliminated after 12 h [110].

Furthermore, a specific scFv was built for the treatment of medullary thyroid carcinoma (MTC), labeled with the radioisotope 131I and tested in nude mice injected subcutaneously with TT cells [111]. Six weeks later, the tumor measured ~1cm, per single-photon emission computed tomography (SPECT) and single-photon emission computed tomography (SPECT-CT). The tumor was visible after 12 h. and a clear image was obtained after 48 h. After three days, the signal was still observed, suggesting that there is good scFv retention in the tumor. In 2017, this same group produced a scFv against anaplastic thyroid carcinoma (ATC) that was labeled with the same radioisotope (131I). It was tested in a murine model inoculated with ARO cells [112]. As soon as the tumor measured over 1 cm^3^, images were obtained by SPECT and SPECT-CT. The result was the same in both tests: radioactivity was initially distributed in most of the organs, but subsequently decreased, except in the tumor tissue. This activity was clearly visible up to 48 h. after the injection.

A scFv of the monoclonal antibody D2B was created against prostate cancer, and it was linked to the prostate-specific membrane antigen (PSMA). The antibody was labeled with 131I and its biodistribution was analyzed with in vivo imaging: the scFvD2B had higher specificity when detecting xenotransplants than the complete antibody [113]. Once its usefulness as a probe was established, scFvD2B was labeled with Xenolight 770 (a fluorophore close to the infrared spectrum) and it was intravenously injected into mice with prostate tumors. Fluorescent molecular tomography (FMT) was used to monitor the tumor´s development in vivo and the fluorescent signal was identified. A peak was observed at 72 h. and these results were confirmed ex vivo [114].

Another research group created a scFv specific for this same antigen; it showed high affinity and was named gy1 [115]. It is internalized in tumor cells through the endosomal–lysosomal route. It was evaluated in a nude mouse model with positive and negative xenotransplants (PC3-PSMA+ and PC3-PSMA−). The scFv was labeled with IRDye800CW, and it was monitored at different moments with fluorescence imaging (FLI) using near-infrared (NIR) conjugates. A fast diffusion was observed and the tumor tissue was clearly detected after 2 h. The peak signal was observed 6 h. after the injection and the signal was almost undetectable after 24 h.

A scFv with receptors for advanced glycation end-products (RAGE) as a target, can help to detect pancreatic cancer in early and metastatic stages because it is overexpressed on the surface of the tumor cells. The anti-RAGE scFv was labeled with a fluorescent molecule (sulfo-Cy5) and it showed great affinity and selectivity for positive RAGE tissues in murine and in vitro human models. The scFv was labeled with copper 64 (64Cu) for detection by PET. Tumors overexpressing RAGE were detected in vivo in a syngeneic mouse model of pancreatic ductal adenocarcinoma (PDAC) [116]. Little accumulation was observed in the tumor, perhaps the result of the rapid renal clearance of the antibody. In future studies, the scFv is expected to be modified into bivalent antibody fragments as a diabody (55 kDa, T1/2 2–5 h) or a minibody (75 kDa, T1/2 5–12 h). This would improve the ratio of target and non-target tissue and would increase imaging sensitivity when detecting the overexpression of RAGE.

There are also biomarkers that regulate different features of neoplasm progression, such as hERG1. It is involved in tumor proliferation, survival, invisibility, and angiogenesis, so it is a good biomolecular marker for the diagnosis and prognosis of cancer [117]. Duranti et al. created a scFv specifically directed against this protein (anti-hERG1 scFv), but the molecule was found to be unstable. It was analyzed in silico, and a cysteine residue was necessary as it was key to the creation of disulfide bridges. However, phenylalanine was detected so it was modified to create an anti-hERG1-cys scFv conjugated with Alexa fluor 450 for possible visualization [118]. This scFv showed good stability and purity and had a great capacity to locate tumors by NIR spectroscopy, as observed in an orthotopic murine model of PDAC. The fluorescent signal was visible 5–10 min after injection, showing a peak signal between 30 and 60 min later. This indicates that it reaches the target cell and is retained in the tumor long enough for its detection by NIR. It was also analyzed in healthy mice and caused no acute or chronic toxicity or cardiac abnormalities.

Mesothelin (MSLN) is another overexpressed molecule in several cancer cells. The specific scFv for this protein was labeled with zirconium 89 (89Zr), and MSLN-positive xenotransplants were successfully observed with PET and CT scans 3 h. after injection. A considerable accumulation of 89Zr was found in the kidneys and the liver since those are the main organs for its elimination; still, there was also fast clearance in the blood, with an average life of 15 min [119].

Zhang et al. created a “tracker” to detect the expression of the vascular cell adhesion molecule-1 (VCAM-1), a transmembrane glycoprotein related to the development of tumors and metastasis. In addition to detecting the protein, the researchers also monitored the healing effects of an IKKβ inhibitor (LY2409881) that induces apoptosis in VCAM-1-positive cells. This tracker consists of the scFv that recognizes VCAM-1 and a bifunctional chelating agent (NOTA-NHS ester), useful for the recovery of the marking molecule; in this case, it was labeled with Gallium 68 (68Ga) [120]. This scFv was successfully used to visualize VCAM-1-positive tumors by PET/CT; the tumor was visualized, and there was rapid clearance from the circulation and high radioactivity in the kidneys. This suggests that the primary clearance route is renal. LY2409881 was proven to bind to VCAM-1, so the link to the scFv was decreased during treatment. This suggests that this scFv could potentially become a personalized patient treatment and an effective monitoring tool.

Since scFvs are rapidly eliminated from the body (average life span: 0.5–2 h) and their affinity is lower due to their monovalence, their retention in the tumor is limited [11]. Therefore, several approaches have been created to modify and confer a superior retention capacity. One of these was reported by Freedman et al., who developed an immunoliposome (~100 nm) containing surface-specific scFvs against Transferrin Receptor 1 (TfR1) and an encapsulated contrast agent for MRI (gadopentetate dimeglumine, gad-d) [121]. The researchers proved its ability to detect lung cancer and carry gad-d to the lung nodes (measuring 100–200 µm) in a murine model.

Another example of a beneficial modification is the generation of more repetitions of the same fragments to increase their capabilities. First, a scFv named MFE23 (28.2 kDa) was specifically developed against carcinoembryonic antigen (CEA, blood, and epithelial tissue marker). It efficiently and specifically localized CEA-positive xenotransplants [122], and a trivalent homotrimer was generated (anti-CEA monomeric MFE23N-trimerbody (scFv-TIE) 3:110 kDa). Each monomer consists of MFE23-scFv fused to the human collagen XVIII trimerization domain of TIE. These were labeled with iodide 124 (124I) for in vivo analysis in PET studies and comparisons between them were conducted. The trimer showed a higher affinity for CEA, a greater accumulation in tumors, and a superior binding capability, in comparison with the monomer [123].

On the other hand, Jugniot et al. generated an anti-thymocyte scFv (Thy1-scFv) differentiation antigen as a fusion protein with an N-terminus sequence that included hexahistidines-3× as purification labels, a Trx label, and an S label for improved solubility. In vivo studies using Thy1-scFv conjugated to an ultrasound contrast agent (MBThy1-scFv) reported an increase in signal intensity in a mouse model of transgenic pancreatic ductal adenocarcinoma (PDAC) versus the non-targeted control. This underscores its potential in the early diagnosis of PDAC (94).

Aside from the diagnostic images, immunoassays such as ELISA have also been developed. They detect biomolecules in blood and urine samples, a great advantage since they may be an effective initial diagnostic tool. Cui et al. developed an assay called BA-ELISA (biotin-avid enzyme-linked immunosorbent assay) based on a specific biotinylated scFv against glycolytic acid (GCA) [124]. This is a human hepatocellular carcinoma (HCC) biomarker found in serum and urine. The average concentration in patients is three-fold higher than that in healthy subjects (11.5 µg/mL and 3.9 µg/mL, respectively). This assay proved to be precise and reliable for the detection of GCA in urine samples, thus promoting early disease detection and greater survival rates. Table 1 summarizes the major scFvs developed for cancer diagnosis.

### 3.2. Cancer Therapy

The affinity and high specificity of antibodies against a specific target and their flexibility upon the addition of functions to the antigen-binding domain, underlie the use of recombinant antibodies and their derivatives in antitumor therapy [126]. There are reports on the great benefits of scFvs resulting from their capability to rapidly penetrate tumor tissue, their high affinity, and their uniform distribution in the tumor, in comparison with whole antibodies that are concentrated in the area adjacent to the tumor [21,127]. No scFv absorption by the kidney has been documented [128]. Various specific scFvs have been developed against cell surface antigens and molecules associated with tumor proliferation, migration, and apoptosis for the management of cancer.

#### 3.2.1. scFvs against Tumor Cell Surface Antigens

Cellular transformation promotes the overexpression of various cell surface antigenic molecules that increase tumorigenesis. Several studies have reported the development of scFvs targeting tumor cell surface antigens, such as mesothelin, a surface antigen in ovarian cancer and mesothelioma; hence, these specific scFvs have also been fused with toxins such as *Pseudomonas* Endotoxin A and the administration of this specific scFv showed a high-binding affinity of the immunotoxin to mesothelin and it was stable for up to 40 h. at 37 °C. Most importantly, it was highly cytotoxic and led to the regression of tumors overexpressing the antigen [129]. Later, Frigerio et al. developed a specific scFv against the prostate-specific membrane antigen (PSMA), an antigen detected in prostate cancer. In vitro assays revealed that the scFv had adequate stability and was tightly bound to the antigen, whereby 40% of the administered scFv was internalized [113]. Another important antigen in the development of prostate cancer is the six-transmembrane epithelial antigen of prostate-1 (STEAP-1), located in intercellular junctions, and overexpressed in all stages of prostate cancer. It plays a role in various cell functions such as proliferation, migration, and metastasis formation. Esmaeili et al. developed a scFv against STEAP-1, a scFv with an inhibitory effect mediated by blocking the union between prostate cancer cells [130]. The use of other antibody fragments such as BiTE, has also led to promising results in the management of this neoplasm. Studies with BiTE anti-CD3 x anti-PSCA and anti-CD3 x anti-PSMA in cancer cell lines have reported specific lysis of close to 50% of cells at a concentration of 30 pmol/mL. These findings underscore how a very small amount of BiTE can promote cell death [131,132]. 

In liver cancer, an anti-AFP scFv (alpha-fetoprotein, a hepatocellular carcinoma marker) combined with administered paclitaxel, resulted in a decrease in the density of tumor vessels and a greater decrease in tumor size in mice when compared with only the administration of paclitaxel [133]. CD176 is a tumor-associated antigen that is specifically expressed on the cell surface of various types of cancer, and that has been found to play a role in the development of metastases. Liu J et al. developed a scFv against this antigen, and it inhibited the adhesion of CD176+ tumor cells to endothelial cells and hepatocytes [134]. Other authors have documented the efficacy of a scFv against the CA125 antigen, an overexpressed protein in various solid tumors. Yu et al. demonstrated that an anti-CA125 scFv specifically recognized the antigen on breast cancer cells [135]. 

We must mention that scFvs have also been shown to efficiently transport and release molecules into the tumor microenvironment in in vitro and in vivo models. In this context, Tong et al. fused the SEB superantigen SEB (Staphylococcal enterotoxin B) to a scFv against MG7 (MG7-scFv/SEB), a protein associated with gastric carcinoma; the administration of MG7-scFv/SEB to rats with gastric cancer resulted in an increase in inflammatory cell infiltrates, the inhibition of tumor cell growth, and an increase in their survival, in comparison with rats treated with saline solution [136]. Likewise, Gattenlöhner et al. fused Pseudomona endotoxin A to a scFv derived from a Fab fragment against the γ sub-unit of the fetal acetylcholine receptor (fAChR) (scFv-ETA). The fAChR is only expressed in rhabdomyosarcoma (RMS) cells after neonate delivery. The use of the scFv-ETA in an in vitro model of RMS eliminated embryonic RMS cells and the intraperitoneal administration of the scFv-ETA in a murine rhabdomyosarcoma model, which delayed the development of the neoplasm [137]. Other examples of carriers of heterologous molecules mediated by a scFv are presented in studies by Wang et al. They generated immunoliposomes that were conjugated to a completely human anti-EGFR scFv that contained doxorubicin and vinorelbine; their results showed that this immunocomplex improved endocytosis and significantly decreased the drugs´ therapeutic dosages in several cell lines of squamous cell carcinoma of the head and neck (SCCHN). It also improved survival in subcutaneous xenograft and orthotopic SCCHN murine models, without evidence of systemic toxicity [138]. Razin et al. developed a specific scFv against the gastrin/cholecystokinin 2 (CCKR2) receptor, that plays a fundamental role in the initiation and development of gastric adenocarcinoma, gastric lymphomas, and gastric neuroendocrine tumors. This scFv showed high-affinity binding to the CCKR2 on cells that overexpressed the marker [97]. 

An anti-CD22 scFv has been recently developed. CD22 is a transmembrane protein specific to B cells and is present in 60–80% of all B-cell lymphomas and leukemias. The anti-CD22 scFv proved to be specific in CD22+ lymphoma cell lines [139]. Likewise, Morali-Kalbolandi et al. developed an anti-CD123 scFv, a molecule that is overexpressed in leukemic stem cells. The anti-CD123 scFv bound and inhibited CD123/IL-3 interactions in an erythroleukemia cell line [140]. 

#### 3.2.2. scFvs against Tumor Growth, Survival, and Proliferation

In cancer, the overexpression of receptors associated to mechanisms that induce growth and proliferation, are an important therapeutic target. Thus, Nickho et al. developed a scFv against the frizzled 7 (Fzd7) receptor, that acts on the dynamics of tumor cell growth. The scFv presented reactivity, inhibited cell growth, and induced apoptosis in a breast cancer cell line [141].

A scFv has also been developed against transferrin receptor 1 that is overexpressed in a broad range of tumors. 3TF12 and 3GH7 are neutralizing human scFvs, originally identified by their ability to bind and rapidly be internalized upon interaction with the human TfR1. These scFvs successfully inhibited the proliferation of neoplastic hematopoietic cell lines such as Jurkat, ERY-1, K562, HL-60 APL, and Raji BL. The bivalent versions, known as F12CH and H7CH, further improved the antiproliferative effect [142], mediated by the inhibition of TfR1 function and iron deprivation. 

Peng et al. reported the development of an “intrabody” (a scFv expressed inside the cell and directed against a particular compartment) named scFv-HAK, against the human TfR1. The scFv-HAK blocked the TfR1 at the level of the endoplasmic reticulum (ER), inducing iron deprivation, inhibiting proliferation, and promoting apoptosis in MCF-7 human breast cancer cells [143].

Another important receptor involved in the proliferation and survival of tumor cells is the epidermal growth factor receptor (EGFR). This protein is overexpressed in colorectal cancer, pancreatic cancer, prostate cancer, lung cancer, and cancers of the head and neck. Veisi et al. used a humanized scFv against EGFR, with the ability to recognize the receptor on epidermoid carcinoma cells [144]. 

*RAS* genes also play an important role in tumor development and progression. The product of the *RAS* gene is a protein known as p21Ras; its neutralization in the cytoplasm could possibly effectively block ras signaling, so Yang et al. developed an anti-p21ras scFv that was extremely immunoreactive in different human cancer tissues, but weakly reactive in normal tissue [145]. Another study that used a recombinant adenovirus carrying the gene for the anti-p21Ras scFv, was conducted in a colorectal cancer xenotransplant model [146] and resulted in the complete elimination of tumor growth. Another novel strategy against colorectal cancer shows that 16 × 133 BiKE (bispecific killer cell engager) specifically binds and activates resting NK cells, inducing degranulation and interferon-γ (IFN-γ) production against CD133+ cells. This BiKE enhances the innate system’s ability and can induce antibody-dependent cell-mediated cytotoxicity and kill tumor cells [147].

**Figure 3:**
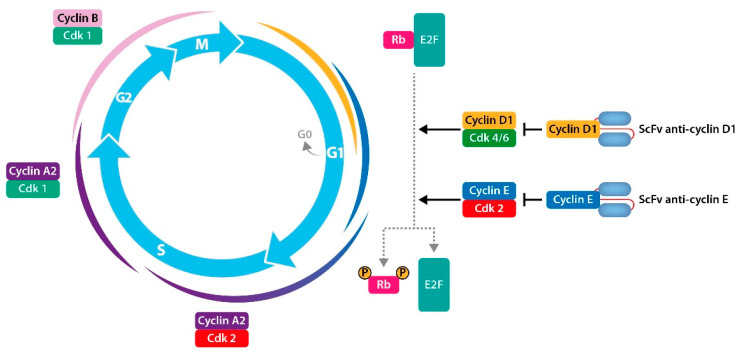
Mechanism of action of intracellular scFv “intrabodies” against cell cycle proteins. Intracellular scFvs are designed with a sequence signaling their retention in the endoplasmic reticulum (ER). Once in the ER, the intrabodies can interact with newly synthesized cyclin E and cyclin D1 proteins, inhibit their function, and thus decrease the levels of phosphorylated retinoblastoma protein, leading to the arrest of the cell cycle in the G1 phase and inducing apoptosis.

Additionally, Wu et al. reported the generation of a scFv against cyclin D1 (named ADκ). Purified ADκ specifically recognized recombinant and endogenous cyclin D1 with high affinity. To block the activity of intracellular cyclin D1, a sequence for signal retention in the ER was added to the ADκ sequence, to encode the ER-ADκ of the anti-cyclin D1 intrabody. Transfection of HepG2 cells with the expression vector encoding ER-ADκ led to the intracellular expression of ER-ADκ, that in turn, caused the union of cyclin D1, the significant arrest of the cell cycle in G1, and apoptosis; all are mechanically linked to a decrease in the levels of intracellular phosphorylated retinoblastoma protein (Rb). Further, ER-ADκ drastically inhibited the growth of subcutaneous hepatocarcinoma (HCC) xenografts in nude mice. These results demonstrate the potential of this therapy against cyclin D on the intrabody, a promising approach to the management of HCC [148]. Strube et al. constructed a scFv derived from anti-cyclin E murine hybridoma cell lines that was managed to be expressed in the cytosol and was directed against the nucleus of breast cancer cells. The stable expression of the anti-cyclin E-scFv inhibited the growth of the breast cancer cell line. Cyclin E is a critical protein in the cell cycle, controlling the regulated progression of normal cells to DNA replication [149]. (Figure 3).

The integrin family plays an important role in cancer angiogenesis; the αvβ3 (ITG αvβ3) integrin sub-type is the main component of the vascular cellular adhesion receptor, since it significantly contributes to invasive angiogenesis and its overexpression promotes tumor growth. Qiu et al. conjugated a scFv-αvβ3 with a molecular peptide, cdGIGPQc, to improve the specificity of the union to integrin αvβ3 in a lung cancer xenotransplant murine model [150].

#### 3.2.3. scFvs against Tumor Migration

Migration is important to cell function and abnormalities in the proteins involved in this process significantly foster carcinogenesis mechanisms while also harboring the potential of becoming a therapeutic target in cancer management. Zhang et al. reported using a scFv that binds to Reg4, a molecule that is part of the gene family derived from human regeneration islets (hReg); it led to an acceleration in tumor growth and resistance to the chemotherapy agent 5-Fluorouracil. This scFv inhibited cell proliferation in a gastric cancer model and curbed the lethal effects of 5-FU [151]. 

A scFv has also been developed against the insulin-like growth factor binding protein 2 (IGFBP2), known to be overexpressed in glioblastoma and associated with increased cellular migration and invasion. Patil et al. showed that this scFv specifically recognized IGFBP2 and induced a decrease in cell migration and invasion in glioblastoma cells [152].

Other groups have documented the generation of a scFv against MUC18, a cell surface receptor known as melanoma cellular adhesion molecule (MCAM) and whose altered expression affects the motility and invasion processes in breast cancer. Mohammadi et al. developed a scFv against MUC18 that inhibited migration by 76% and cellular invasion by 67% in MUC18+ cells [153]. 

The construction of scFvs against gelatinases has also been documented; these are metalloproteinases of the extracellular matrix (MMP), and play a pivotal role in tumor angiogenesis, invasion, and the development of metastasis. The inhibition of these molecules could result in the suppression of these tumor development processes. Gao et al. developed an anti-gelatinase scFv fused with the apoprotein, lidamidine (LDP), that when combined with Endostar, an angiogenesis inhibitor, curbed tumor growth in a xenotransplant murine model of human hepatoma [154].

#### 3.2.4. scFvs Can Induce Tumor Apoptosis

The induction of apoptosis is a process inherent to cellular injury and its promotion and recovery in different types of tumors is a promising therapeutic alternative to eliminate cancer. Various scFvs have been developed with this ultimate purpose, as reported by Amoury et al., who developed a scFv against the epithelial cellular adhesion molecule (EpCAM). This molecule is associated with a poor prognosis when overexpressed. The scFv was genetically fused to a granzyme B mutant that activates many apoptosis signaling pathways; the intravenous administration of the scFv in a triple-negative breast cancer (TNBC) xenotransplant murine model, induced apoptosis and inhibited tumor growth [155]. 

The scFvs have also been used to direct liposomes containing antitumor molecules to the tumor microenvironment. As an example, the liposome–scFv anti-TfR1 5E9 complex used as a system to administer low-toxicity systemic genes selectively directed against tumor cells, can be selectively directed against tumor cells by carrying molecules such as the tumor suppressor gene p53. The administration of this liposome–antiTfR1-*p53* gene in combination with docetaxel improved survival in a xenotransplant murine model of human breast cancer metastasis (154). Another study conducted by Xu et al. documented that cationic immunoliposomes that incorporate a variable fragment (Fv) against the transferrin receptor (TfRscFv) to release the p53 gene, improved the penetration of these complexes into prostate cancer cells [156]. The intravenous administration of immunoliposomes with TRscFv to mice with xenotransplants of carcinomas of the head and neck, demonstrated consistent transport to the tumor site and efficient expression of the tumor suppressor gene p53 in comparison with liposomes without an associated ligand. In another study that followed a different bioconjugation method, the intravenous administration of immunoliposomes conjugated with polyethylene glycol and a TfRscFv, resulted in an increase in genetic expression than those mediated with the same system but without PEG. Furthermore, the increased expression of exogenous genes in a mouse xenotransplant model of human pancreatic PANC-1 cells and the low hepatic expression of p53 suggest that the PEG-conjugated systems are preferentially directed to the tumor [157]. Table 2 summarizes the major sfFv developed for cancer therapy. 

## 4. scFvs in Cancer Therapy Clinical Trials

Several studies have been developed to improve the specificity and efficacy of scFvs. Some of these molecules are aimed at a specific target and have been tested in clinical trials (Table 3).

The selective administration of therapeutic molecules against primary and metastatic tumors is ideal for the development of an efficient treatment against cancer. Some clinical trials have particularly focused on hematological cancer therapy; for example, modified T cells (CAR-T) express a scFv on their surface, these CAR-T cells targeting the B-cell marker CD19 have shown unprecedented response rates in treating refractory B cell malignancies and became the first genetically modified cell-based therapy to receive US Food and Drug Administration approval [160].

Recently, the FDA approved a new treatment against metastatic uveal melanoma, the IMCgp100 (tebentafusp-tebn), a specific biological therapy that incorporates a T cell receptor (TCR) specifically designed against a peptide antigen derived from protein gp100 on the HLA-A2 of melanoma cells. The TCR is fused with an anti-CD3 scFv that recruits and activates non-specific melanoma T cells (killer T cells) against neoplastic cells (NCT03070392).

Other clinical trials have used the scFv against transferrin receptor 1 (TfR1). These scFvs are coupled to lysosomes carrying the p53 gene, thus creating a complex named SGT-53. The phase Ib clinical trial evaluated the safety profile of the complex when combined with docetaxel in advanced solid tumors (NCT00470613) [161,162]. The combination was adequately tolerated but its clinical effects were considered moderate. It is currently under evaluation in phase II trials [163]. Likewise, the nanocomplex SGT-94 is also undergoing evaluation in a clinical trial; this complex is constituted by an anti-TFR1-liposome scFv and a truncated variant of the RB gene (RB94). The phase I study results in metastatic genitourinary cancer using intravenous SGT-94, showed minimal side effects, but the clinical activity led to complete or partial tumor remission in patients, with a 2.4-mg dose (NCT01517464) [164]. 

BiTEs are new molecules used in cancer therapy against solid and hematological tumors. They are created by linking two scFvs, one bound to the lymphocyte and the other to tumor-associated antigens. They have proven to possess antitumor properties; for instance, MT110 (Solitomab) targets EpCAM and CD3, and this BiTE inhibited the development of metastases and tumor growth in advanced solid tumors (colorectal cancer, breast cancer, prostate cancer, and ovarian cancer) (NTC00635596). Blinatumomab (MT-103) is the most advanced BiTE that has been evaluated in clinical trials and was recently approved by the FDA in its Accelerated Approval Program; it is used in the treatment of non-Hodgkin lymphoma and acute lymphoblastic leukemia. Final approval will depend on the validation and confirmation of the clinical trial´s results. This BiTE is targeted against CD3+ and CD19+ lymphocyte malignancies. In Phase I, cytotoxicity against B lymphocytes was demonstrated and its clearance profile and secondary effects were evaluated in Phase II; children have been recruited for Phase III [165,166,167]. Other BiTEs have shown efficient antitumor activity; for example, the MT111 BiTE targets the carcinoembryonic antigen and glycoproteins pertaining to the immunoglobulin superfamily and that are detectable in a wide range of solid tumors [168,169]. Another example is BAY2010112 (Pasotuxizumab), a BiTE that recognizes CD3 and PSMA and is used to treat prostate cancer. Phase I studies have confirmed its safety profile and its dose-dependent clinical activity (NTC01723475).

Other clinical trials evaluating scFvs have been conducted with Vicinium and Proxinium. The active ingredient in these compounds is VG4-845, a recombinant fusion protein produced in *E. coli* and expressing a humanized-specific scFv against the EpCAM antigen bound to a bacterial toxin fragment. Once bound to the EpCAM antigen on the surface of the epithelial cells of tumors, this compound is internalized via an endocytic pathway. The other molecule is separated and induces cell death by irreversibly blocking protein synthesis. Proxinium was tested in head and neck squamous cell carcinoma to evaluate its pharmacokinetics and immunogenicity. Patients received two rounds of five intratumoral VB4-845 injections daily (20, 40, 80, 130, 200, or 280 µg). The maximum tolerated dose was 280 µg/daily/5 days. The most common adverse events were pain due to the intra-tumoral injection and reversible hepatic enzyme elevations. Still, it was well tolerated and will be evaluated in other studies [170]; it is currently undergoing Phase II (NCT00272181) and III (NCT00412776) evaluations. This same molecule has been studied in other types of cancer, such as invasive bladder carcinoma, in which the intravesical administration of Vicinium could potentially provide an alternative to cystectomy. This study is currently in Phase III (NCT02449239).

## 5. Limitations

Despite the advantages inherent to scFvs, they also possess drawbacks that may limit their therapeutic potential. 

*Stability.* In comparison with mAb, scFvs are less stable due to their tendency to form aggregates under thermal stress conditions [171], because they lack the Fc domain [172]. Several groups have developed tools to prevent scFv aggregation and improve their stability. Curtis et al. were able to decrease the propensity of scFv to aggregate by replacing arginine residues, that alter the specificity and selectivity of the antigen–antibody complex, with lysine [173]. This modification could be helpful in the design of scFvs to generate stabilizing scFv mutations, albeit at the expense of the specificity and selectivity of antigen–antibody interactions. This could be prevented if mutations are made in residues located far from the binding site [174].

*Half-life.* The small size of scFvs is a beneficial property because it increases tissue permeability, especially when penetrating tumors in therapeutic settings [172,175]. However, their half-life is also limited (<1 day versus 3 weeks with full-size IgG) because their low molecular weight precludes their clearance by glomerular filtration (65 kDa) [9]. Some strategies have been developed to increase their half-life, such as combining the scFv with other molecules. One of the most common resources is the addition polyethylene glycol molecules as a stabilizer to decrease their clearance rate from blood; another strategy is using long-lived serum proteins such as albumin [176], that also increases the half-life of circulating scFvs. However, these fusions may compromise some of the scFv´s characteristics as a result of their increase in size [172], such as tissue permeability, one of their most relevant features [9].

*Expression and production.* High scFv expression is a challenge due to the fragments´ tendency to aggregate as a result of the hydrophobic interaction of the VH and VL domains. To obtain adequate expression, several methods may improve the stability of the generated fragments and require the modification of specific residues within its structure as well as random mutagenesis [177]. Some authors have also suggested scFv humanization by replacing the hydrophobic amino acids with hydrophilic residues to prevent their accumulation [178]. Unfortunately, scFv cannot be efficiently expressed in yeast expression systems since appropriate folding is precluded by hydrophobicity and requires additional steps such as chaperone expression that could curb this limitation [179]. Bacterial expression systems, however, appear to be an option to obtain an optimal yield of antibody fragments, while also decreasing production costs [180].

*In vivo administration.* In view of the scFvs instability and half-life, their in vivo application would require higher dosing or more frequent administration to obtain the desired effect; to counteract this limitation, researchers have suggested liberating the scFvs with viral vectors, thus avoiding the need for high doses and constant administration, but still obtaining a sustained scFv concentration [177,181].

*Routes of administration.* To date, the optimal route of scFv administration in humans remains uncertain. In murine preclinical models of diseases other than cancer [9,181], intravenous administration has been posited and even the intracranial route has been suggested, both yielding good results; however, their use in human treatments will require less invasive routes of administration and there are undergoing studies evaluating the intramuscular and intranasal routes, with apparently promising results [9]. 

## 6. Conclusions

Recent advances in cancer immunology and recombinant DNA technology have allowed the successful use of antibody derivatives such as scFvs for the diagnosis and selective treatment of cancer. This review documents the improvements in the properties of scFvs in terms of antigen stability, avidity or affinity, and specificity. These have improved scFvs as a diagnostic tool when combined with a wide range of molecules that are easily identified with modern imaging methods, such as MRI, SPECT, CTI, and PET. The ability of scFvs to recognize tumor-associated antigens, oncogenes, and tumor suppressor genes has been clearly documented. Studies have explored their ability to carry several molecules, including bacteria-derived toxins, immunomodulatory molecules, and chemotherapeutic agents. Breakthroughs in clinical trials have demonstrated that scFvs are safe molecules and with great antitumor activity when incorporated into CART or BiTE systems. To date, these molecules represent a novel alternative with great potential in the diagnosis and therapy against cancer, although further optimizations are required to render scFvs fully functional.

## Figures and Tables

**Table 1:** scFv as a diagnostic tool in cancer.

Target scFv	Type of Cancer	Diagnostic Method	Reference
Epithelial cell adhesion molecule (EpCAM)	Adenocarcinoma and squamous cell carcinomas (colorectal)	MRI	[105]
Epidermal growth factor receptor (EGFR)	Non-small cell lung cancer	MRI	[106]
Tissue factor (TF)	Solid tumors such as gastric, pancreatic, and brain cancer	IVIS in vivo imaging system	[107,109]
Medullary thyroid carcinoma	Medullary thyroid carcinoma	SPECT and SPECT-CT	[111]
Anaplastic thyroid carcinoma	Anaplastic thyroid carcinoma	SPECT and SPECT-CT	[112]
Prostate-specific membrane antigen (PSMA)	Prostate cancer	Fluorescent molecular tomography (FMT)	[114,115]
Prostate-specific membrane antigen (PSMA)	Prostate cancer	Fluorescence imaging (FLI) using near infrared (NIR)	[115]
Receptor for advanced glycation end-products (RAGE)	Pancreatic cancer	PET	[116]
hERG1	Various neoplasms	Near-infrared (NIR) spectroscopy	[117,118]
Mesothelin (MSLN)	Various neoplasms	PET/CT	[119]
Vascular cell adhesion molecule-1 (VCAM-1)	Various neoplasms	PET/CT	[120]
Transferrin receptor	Lung tumors	MRI	[121]
Carcinoembryonic antigen (CEA)	Adenocarcinomas	PET	[122]
Anti-thymocyte differentiation antigen	Pancreatic ductal adenocarcinoma	Ultrasound	[124]
Glycolytic acid (GCA)	Human hepatocellular carcinoma	ELISA	[125]

Abbreviations: MRI, magnetic resonance imaging; SPECT, single-photon emission computed tomography; CT, computerized tomography; PET, positron emission tomography.

**Table 2:** scFvs as cancer therapy.

Target	Type of Cancer	Reference
**scFv against tumor-cell surface antigen**		
scFv/Mesothelin	Epidermoid cervical carcinoma	[129]
Prostate-specific membrane antigen (PSMA)	Prostate cancer	[113]
Alpha-fetoprotein (AFP)	Hepatocellular carcinoma	[133]
CA125	Breast cancer	[135]
Six-transmembrane epithelial antigen of the prostate (STEAP-1)	Prostate cancer	[130]
CD176	Gastric and colorectal cancer	[134]
MG7-scFv/SEB	Gastric cancer	[136]
fAChR scFv/ETA	Rhabdomyosarcoma	[137]
CD22	Lymphoma	[139]
Cholecystokinin-2/gastrin receptor (CCKR2)	Gastric adenocarcinoma	[97]
Epidermal growth factor receptor III (EGFRvIII)	Glioblastoma	[144,158]
CD123	Erythroleukemia	[140]
**scFv against tumor growth, survival, and proliferation**		
Frizzled class receptor 7 (Fzd7)	Breast cancer	[141]
Transferrin receptor 1 (TfR1)	Squamous cell carcinoma and hematopoietic neoplasms	[138,142,156,159]
p21Ras	Colorectal cancer	[146]
Cyclin D1	Hepatocellular carcinoma	[148]
Cyclin E	Breast cancer	[149]
Integrin alphavbeta3 (ITG αvβ3)	Lung cancer	[150]
**scFv against tumor migration**		
Reg4	Gastric cancer	[151]
Insulin-like growth factor binding protein-2 (IGFBP2)	Glioblastoma	[152]
MUC18	Breast cancer	[153]
Lidamidine apoprotein (LDP)	Hepatocellular carcinoma	[154]
**scFv inducing tumor apoptosis**		
Epithelial cell adhesion molecule (EpCAM)	Triple-negative breast cancer (TNBC)	[155]
p53	Solid tumors	[159,160]

**Table 3:** Clinical trials of therapeutic agents against cancer with scFvs.

Format	Type of Cancer	Clinical Trial Identifier	Phase	Start Year
T cells modified with RNA anti-cMET CAR	Malignant melanoma Breast cancer	NCT03060356	Phase I	2017
CAR-T-BCMA	Multiple myeloma	NCT02546167	Phase I	2015
CAR-20/19-T cells	Acute lymphoblastic leukemia	NCT04049383	Phase I	2019
CAR-20/19-T cells	Non-Hodgkin lymphoma Chronic lymphocytic leukemia	NCT03019055	Phase I	2017
IMCgp100 (tebentafusp-tebn)	Malignant melanoma	NCT03070392	Phase II	2010
Blinatumomab/MT103/MEDI-538	Non-Hodgkin lymphoma Acute lymphocytic leukemia	NTC02101853 NTC02003222	Phase III	2013
BAY2010112/AM112 (Pasutuxizuab)	Prostate cancer	NTC01723475	Phase I	2012
MT-103/scFv-tandem scFv fused with a linker	Non-Hodgkin lymphoma, acute lymphoblastic leukemia	NCT00274742, NCT00538096, NCT01471782, NCT00676871, NCT02101853,	Phases I, II, III	2000
MT-110/scFv- tandem scFv fused with a linker	Solid tumors	NCT00635596	Phase I	2006
MT-111/scFv-tandem scFv fused with a linker	Gastrointestinal tumors, adenocarcinomas	NCT01284231	Phase I	2009
BAY2010112/scFv-tandem scFv fused with a linker	Prostate cancer	NCT01723475	Phase I	2012
Anti-EpCAM ScFv Vicinium VB8-845	Bladder cancer	NCT02449239	Phase III	2015
Anti-EpCAM ScFv Proxinium VB8-845	Squamous cell carcinoma of the head and neck	NCT00272181 NCT00412776	Phases II and III	2006
Plasmid DNA p53 gene encapsulated in scFv liposome	Central nervous system tumors	NCT03554707	Phase I	2018
Plasmid DNA p53 gene encapsulated in scFv liposome	Solid tumors	NCT02354547	Phase I	2015
Plasmid DNA p53 gene encapsulated in scFv liposome	Metastatic pancreatic cancer	NCT02340117	Phase II	2015
Plasmid DNA p53 gene encapsulated in scFv liposome	Recurrent glioblastoma	NCT02340156	Phase II	2015
Plasmid DNA p53 gene encapsulated in scFv liposome	Solid tumors	NCT00470613	Phase I	2007
RB94 gene encapsulated in scFv liposome	Solid tumors	NCT01517464	Phase I	2012
